# Identification of brain antigens recognized by autoantibodies in experimental autoimmune encephalomyelitis-induced animals treated with etomoxir or interferon-β

**DOI:** 10.1038/s41598-018-25391-y

**Published:** 2018-05-04

**Authors:** Anne Skøttrup Mørkholt, Kenneth Kastaniegaard, Michael Sloth Trabjerg, Gopana Gopalasingam, Wanda Niganze, Agnete Larsen, Allan Stensballe, Søren Nielsen, John Dirk Nieland

**Affiliations:** 1 Department of Health Science and Technology, Aalborg University, Aalborg, Denmark; 2 Department of Biomedicine, Aarhus University, Aarhus, Denmark

## Abstract

Multiple sclerosis (MS) is a neurodegenerative autoimmune disease, where chronic inflammation plays an essential role in its pathology. A feature of MS is the production of autoantibodies stimulated by an altered-peptide-ligand response and epitope spreading, resulting in loss of tolerance for self-proteins. The involvement of autoantibodies in MS pathogenesis has been suggested to initiate and drive progression of inflammation; however, the etiology of MS remains unknown. The effect of etomoxir and interferon-β (IFN-β) was examined in an experimental-autoimmune-encephalomyelitis (EAE) model of MS. Moreover, the impact of etomoxir and IFN-β on recognition of brain proteins in serum from EAE rats was examined with the purpose of identifying the autoantibody reactivities involved in MS. Animals treated with etomoxir on day 1 exhibited a statistically significantly lower disease score than animals treated with IFN-β (on day 1 or 5) or placebo. Etomoxir treatment on day 5 resulted in a significantly lower disease score than IFN-β treatment on day 1. After disease induction antibodies was induced to a broad pallet of antigens in the brain. Surprisingly, by blocking CPT1 and therewith lipid metabolism several alterations in the antibody response was observed suggesting that autoantibodies play a role in the EAE animal model.

## Introduction

Multiple sclerosis (MS) is a chronic inflammatory disease of the brain and spinal cord that results in demyelination, neurodegeneration and axonal loss^1^. To date, no cure for MS has been identified, and although treatment for relapse-remitting MS focuses on reducing disease activity and progression, no treatment for the progressive types of MS has been identified^2,3^. One of the first-line therapies for MS is interferon-β (IFN-β), which functions by redirecting immunological responses from pro-inflammatory to anti-inflammatory T cell responses^4^. However, immunomodulatory treatments do not show long-term effects and have no effect on patients entering the progressive phase of MS^2,4,5^. B cells are another potential target for treating MS, and several types of anti-CD20 treatments have shown efficacy in clinical trials^6^. Ocrelizumab is a new recombinant humanized antibody that targets CD20 antigen expressed on B cells and has shown efficacy in phase 3 trials for treatment of both relapse-remitting MS and primary progressive MS^7,8^.

## Multiple sclerosis as an autoimmune disease

The etiology of MS remains unknown; however, environmental triggers in genetically susceptible individuals have been proposed as a model^1,9,10^. T cells are thought to be activated in the periphery through various mechanisms, such as molecular mimicry, by-stander activation and exposure to bacteria present in the colon, and then to traffic to the central nervous system with activated B cells and monocytes. For more than two decades, this hypothesis has been proposed as the framework for understanding and treating MS as an autoimmune disease^9^. However, studies indicate that the T cell biology in MS is complex and that several T cell types play a role in MS pathogenesis^11–13^.

In recent years, the involvement of B cells and autoantibodies in MS has been re-examined, and they are both thought to play a central role in MS pathogenesis due to a correlation between disease progression and synthesis of intrathecal antibodies known as oligoclonal bands^9,14–17^. Nevertheless, limited knowledge is available about the antibody-antigen response in the brain and its role in disease induction and progression^14,18,19^. However, recent studies found that treatment with ocrelizumab, which functions by depleting CD20-expressing B cells, had beneficial effects on primary progressive MS and thereby reinforced the contribution of B cells to MS pathogenesis^8^. Specific posttranslational modifications have been shown to result in the loss of tolerance to the modified proteins, thus initiating antibody recognition^20,21^. A candidate for initiation of such an autoimmune response is a posttranslational modification termed citrullination, which naturally occurs on a myelin protein known as myelin basic protein (MBP). Citrullination is highly immunogenic, and antibodies against citrulline proteins are already used as diagnostic biomarkers in rheumatoid arthritis^20,21^. However, treating relapses with immunomodulatory therapies does not stop progression towards neurodegeneration and axonal loss, and as this effect indicates that the causes of lesions and neurodegeneration involve other mechanisms, key questions remain unanswered. Mechanisms underlying disease progression are proposed to involve an interaction between oxidative stress, mitochondrial dysfunction, energy deficit and ion channel dysfunction, which are also suggested to be involved in neurodegeneration in Alzheimer’s disease (AD), Parkinson’s disease (PD) and amyotrophic lateral sclerosis (ALS)^22,23^.

## Multiple sclerosis as a consequence of metabolic dysfunction

The traditional view of MS as a chronic inflammatory autoimmune disease is controversial. Alternatively, MS has been proposed to be a consequence of lipid metabolism dysfunction as well as mitochondrial dysfunction^24^. Studies have supported this hypothesis, as they have shown impaired glucose metabolism in MS^25,26^. Reduced glucose metabolism indicates a shift in metabolism towards lipid metabolism, which is consistent with previous reports of decreased lipid levels in MS lesions^27,28^. In addition, research has shown that T cells exposed to stress also induce a switch from glucose to lipid metabolism as an energy source, which underpins the importance of lipid metabolism in immune cells^29^. Carnitine palmitoyl transferase 1a (CPT1a) is a key enzyme involved in lipid metabolism, as it catalyzes the conversion of acyl-CoA (which is mitochondrial membrane-impermeable) into acyl-carnitine (which is mitochondrial membrane-permeable)^29^ [Mørkholt *et al*., submitted for publication]. This indicates that CPT1a serves as a rate-limiting enzyme in beta-oxidation^29,30^. CPT1a expression was found to be upregulated in MS lesions of the spinal cord^31^. Additionally, mutations in CPT1a resulting in either 22% activity or a dysfunctional molecule were found in two human populations in Canada, namely, the Inuits and Hutterites, respectively^32–36^. The incidence of developing MS was found to be 1/50,000 for the Inuits and 1/1100 for the Hutterites, which is rather low compared to the incidence of 1/350 in the Canadian population^37,38^. These findings suggest a role of CPT1a mutations in protecting against the development of MS. Although the particular mechanism involved in the role of CPT1 in MS is unknown, it can be hypothesized that decreased CPT1 activity plays a protective role against mitochondrial dysfunction, as CPT1 plays an essential role in energy production from lipids, which thereby results in diminished production of reactive oxygen species (ROS). Furthermore, ROS and reactive nitrogen species (NOS) are both thought to be mediators involved in demyelination and axonal injury in MS and experimental autoimmune encephalomyelitis (EAE)^39,40^.

Chemical blockage of CPT1 by etomoxir enables a shift in metabolism that favors glucose metabolism rather than lipid metabolism as an energy source^41–44^ [Mørkholt *et al*., submitted for publication]. This CPT1 antagonist inhibits formation of acyl-carnitine, which is necessary for the transport of acyl-CoA into the mitochondria^41^. CPT1 inhibition presents a new treatment strategy that aims at both metabolism and immune system rather than focusing solely on inflammation as a therapeutic target. Studies by Shriver *et al*. and Mørkholt *et al*. supported this new hypothesis by showing that etomoxir treatment resulted in decreased disease activity and inflammation and increased remyelination in an EAE mouse model characterized by the expression of hallmarks of MS such as demyelination and inflammation^11,29^ [Mørkholt *et al*., submitted for publication]. This suggests that etomoxir is a novel potential treatment for MS.

This study aimed to investigate the efficacy of etomoxir and IFN-β treatment on the autoantibody response in EAE-induced and healthy animals using Western blot analysis and to identify and examine the levels of autoantibody reactivity for autoantigens recognized by these autoantibodies by immunoprecipitation and mass spectrometry.

## Results

### Disease score in an experimental autoimmune encephalomyelitis model

**Figure 1:**
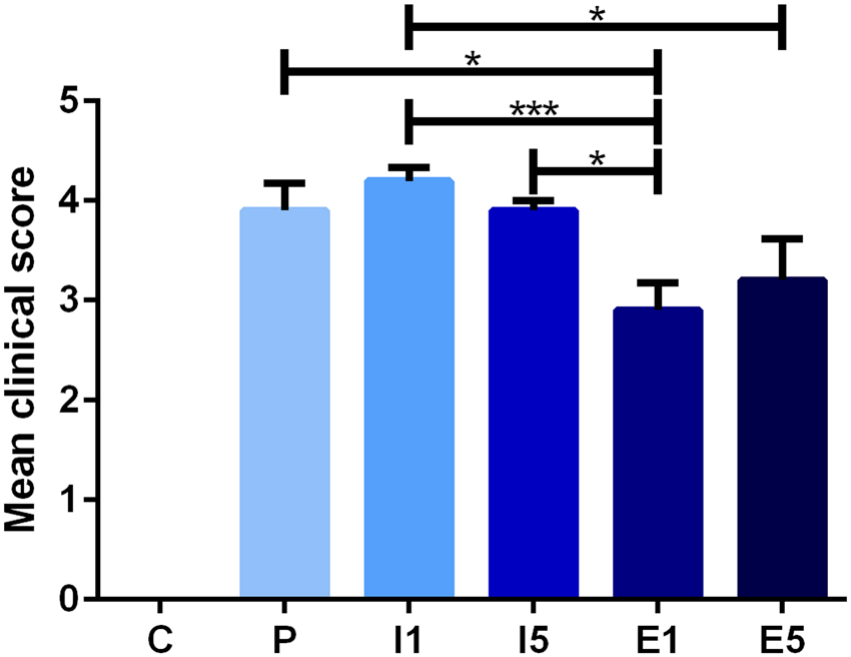
Mean clinical score for animals in the EAE model; control (C, n = 5), placebo (P, n = 10), IFN-β on day 1 (I1, n = 10), IFN-β on day 5 (I5, n = 10), etomoxir on day 1 (E1, n = 10) and etomoxir on day 5 (E5, n = 10). All data are presented as the mean ± SEM. Results from unpaired t-tests showed a statistically significant difference in the disease scores between E1 and I1, I5, and placebo. Treatment on E5 resulted in a significantly lower disease score than that on I1. Number of asterisks indicates level of statistical significance (*p < 0.05 and ***p < 0.001).

The impacts of the two treatments (etomoxir and IFN-β) and two treatment time points (days 1 and 5) were compared with those of the placebo treatment and control groups (Fig. 1). There were statistically significant differences in the disease score between animals treated with etomoxir on day 1 and animals treated with IFN-β on days 1 and 5 and placebo (p < 0.05 and p < 0.001). Moreover, animals receiving etomoxir on day 5 exhibited a significantly lower disease score than animals treated with IFN-β on day 1 (p < 0.01).

### Detection of autoantibodies against brain proteins

**Figure 2:**
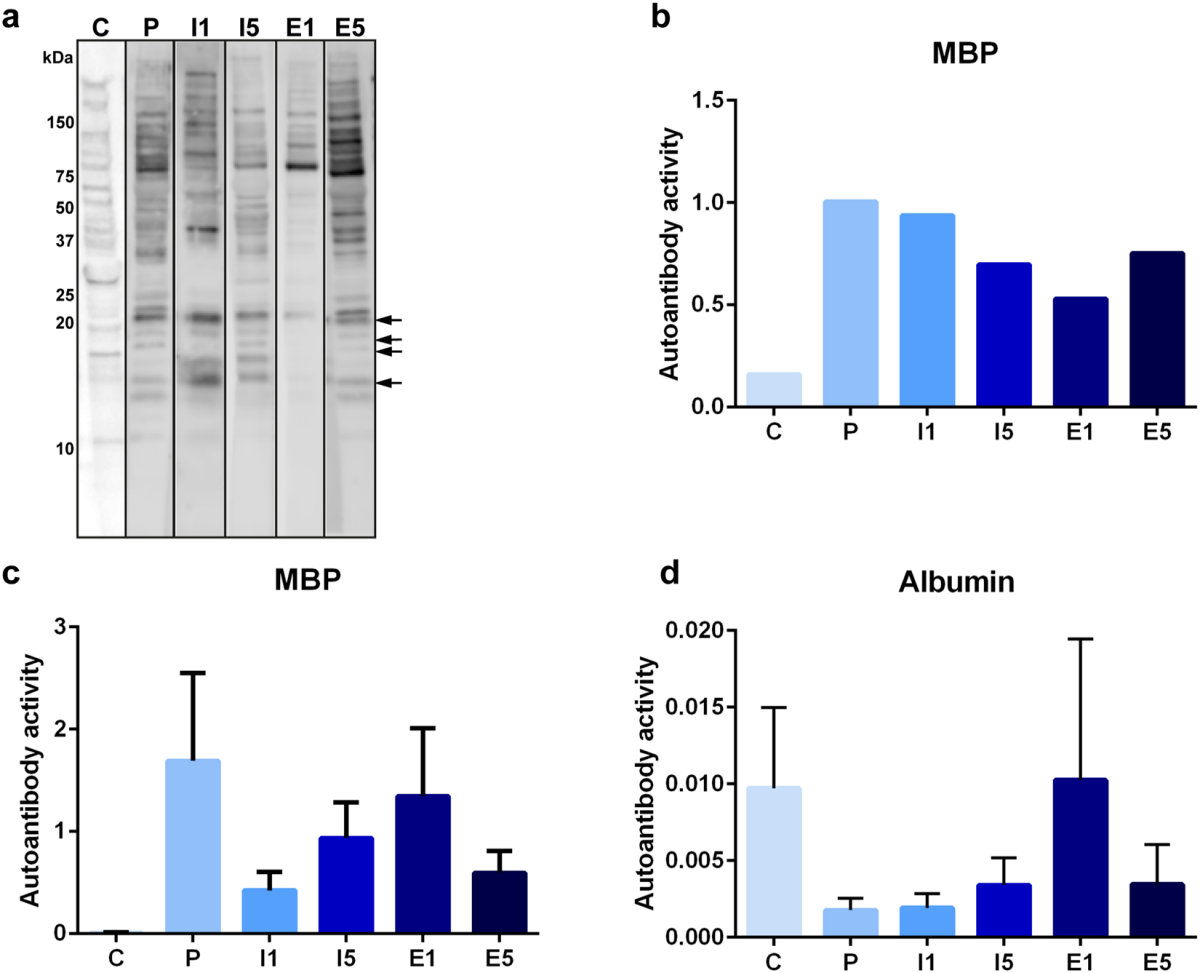
Western blots showing expression of brain antigens recognized by serum-derived antibodies; control (C), placebo (P), IFN-β on day 1 (I1), IFN-β on day 5 (I5), etomoxir on day 1 (E1) and etomoxir on day 5 (E5). Protein marker positions are indicated to the left (kDa). MBP bands are marked with arrows. Serum samples are derived from the same experiment. Each blot represents a sample from the different treatment groups, and blots were cropped and processed in parallel (**a**). Indirect ELISA showing the concentration (µg/ml) of serum-derived autoantibodies towards MBP in the presented blots (**b**). Indirect ELISA showing the concentration (µg/ml) of serum-derived autoantibodies towards MBP (**c**) and albumin (**d**) in all the animals. Data are presented as the mean ± SEM.

Western blot analysis was performed to investigate the immunoglobulin G (IgG) antibody response against brain self-antigens in EAE-induced rats receiving placebo, IFN-β on day 1 or 5, or etomoxir on day 1 or 5 (Fig. 2). Autoantibodies against brain antigens were detected in all animals, and interestingly, EAE- induced rats treated with etomoxir on day 1 showed weaker antigen binding than the other treatment groups. All six groups revealed expression of MBP (Fig. 2a). The autoantibody response to MBP was validated by ELISA (Fig. 2b and c).

### Detection of autoantigens recognized by autoantibodies in the brain

**Figure 3:**
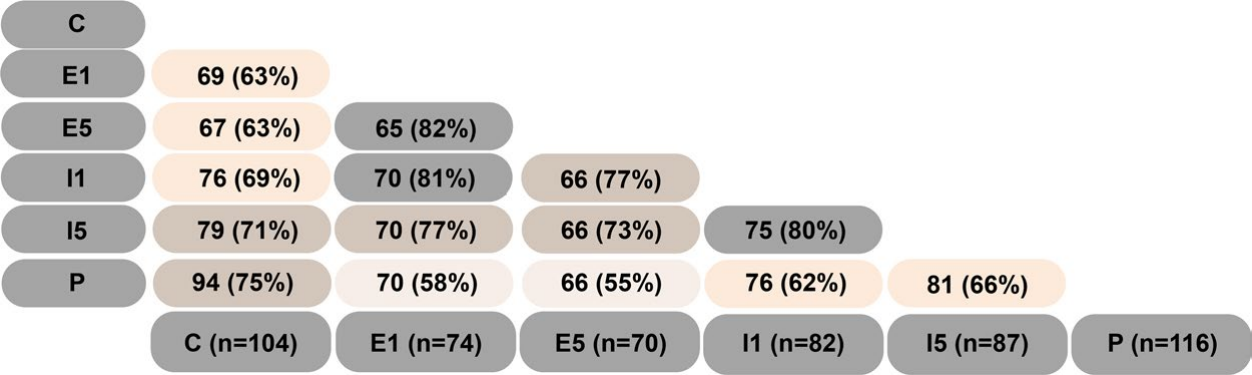
Venn diagram of the immunoprecipitated proteins identified using mass spectrometry. Number of co-identified proteins corresponding to number of autoantibodies and percentages of similarity are shown in the treatment groups; control (C), placebo (P), IFN-β on day 1 (I1), IFN-β on day 5 (I5), etomoxir on day 1 (E1) and etomoxir on day 5 (E5).

To investigate detection of antibodies to brain proteins further, immunoprecipitation was performed, and precipitated proteins were identified and quantified using label-free mass spectrometry. A Venn diagram of the identified proteins showed a high similarity in the number of precipitated proteins between groups (Fig. 3). The highest number of detected proteins in a sample was 123; of these proteins, seven were directly related to IgG (these proteins were discarded because they could have originated from serum antibodies), and 116 were suggested as self-antigens, including MBP and myelin proteolipid protein (PLP).

Interestingly, certain antigens in the control, placebo and treatment groups were highly similar; the lowest similarity percentage was 55%. The differences in the relative abundance, which potentially represents the antibody response, between the treatment groups and the placebo and control groups were investigated.

**Figure 4:**
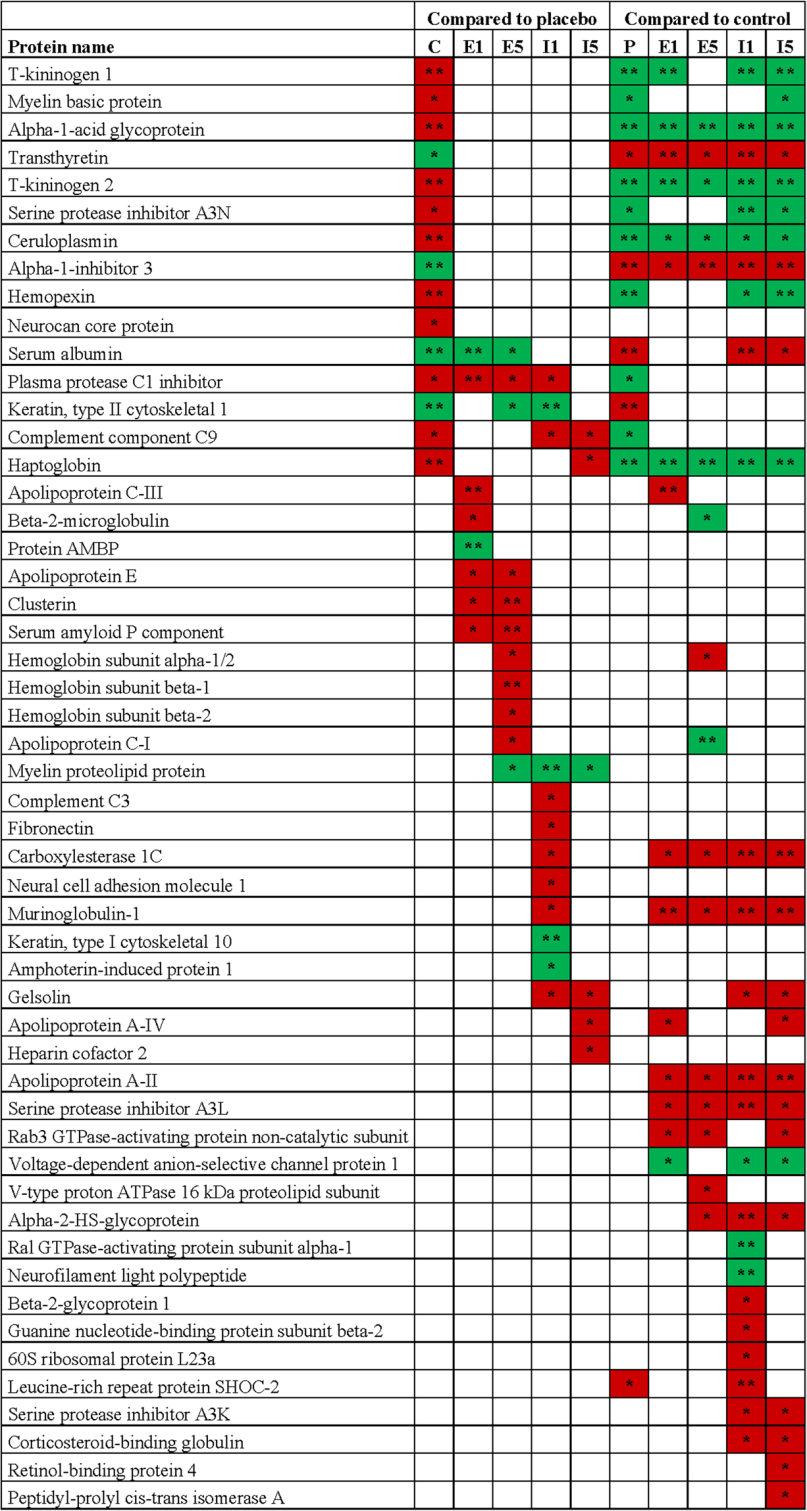
Significant differences in the relative quantification between the treatment groups and the placebo (P) and control (C) groups. Treatments: E1 = etomoxir on day 1; E5 = etomoxir on day 5; I1 = IFN-β on day 1; I5 = IFN-β on day 5. SD under 2.0 on log2 scale.  significantly increased or  significantly decreased by at least a factor of 2. Number of asterisks indicates level of statistical significance (*p < 0.05 and **p < 0.01). More information can be found in Supplementary Figure S1.

There were several significant differences in the quantification of autoantibody reactivity for antigens between the treatment groups and the placebo and control groups (Fig. 4, Supplementary Figure S1).

**Figure 5:**
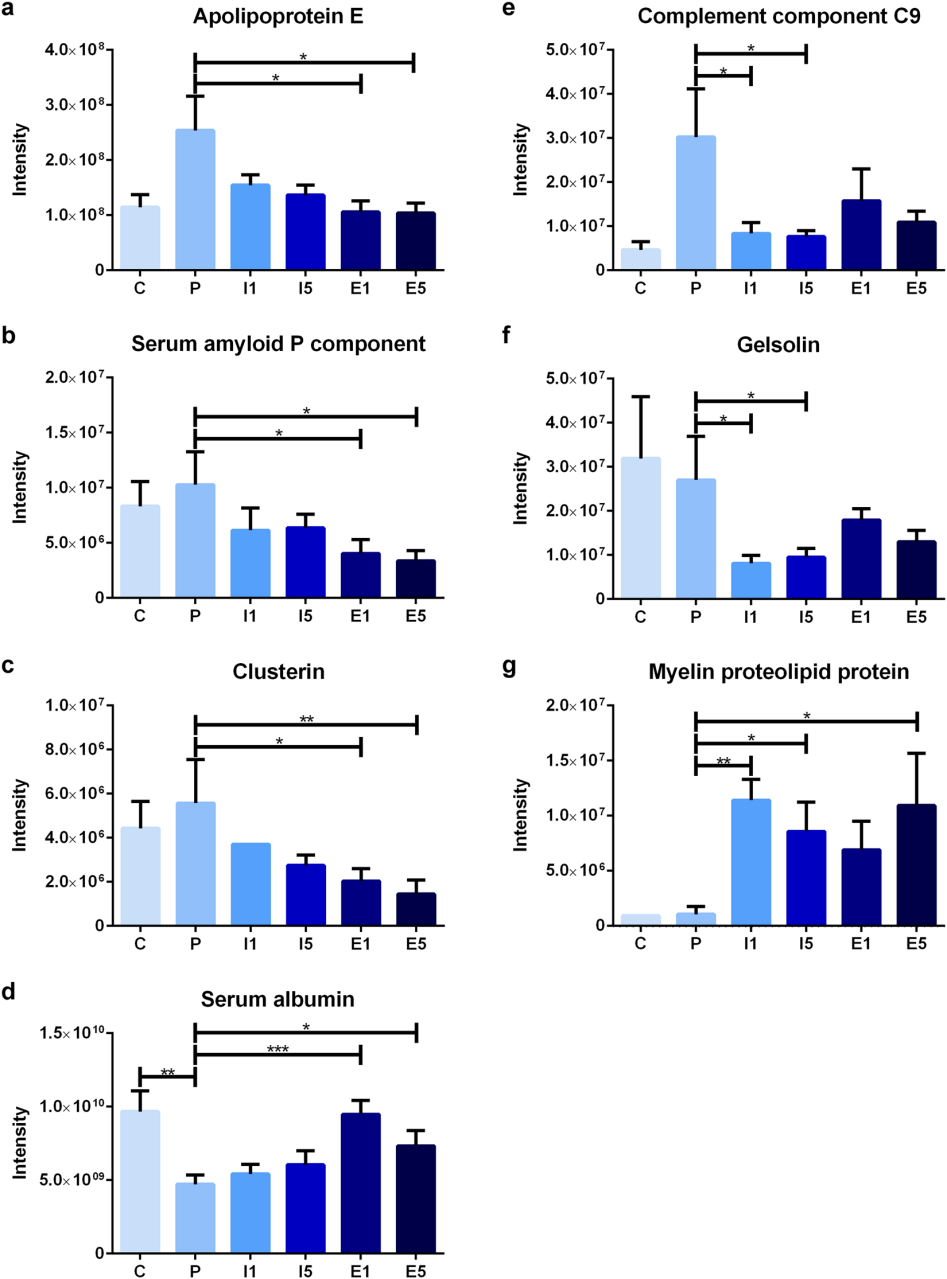
Intensity of immunoprecipitated protein for all groups; apolipoprotein E (**a**), serum amyloid P component (**b**), clusterin (**c**), serum albumin (**d**), complement component C9 (**e**), gelsolin (**f**) and myelin proteolipid protein (**g**); control (C, n = 5), placebo (P, n = 10), IFN-β on day 1 (I1, n = 10), IFN-β on day 5 (I5, n = 10), etomoxir on day 1 (E1, n = 10) and etomoxir on day 5 (E5, n = 10). All data are presented as the mean ± SEM. Results from the unpaired t-tests showed statistically significant differences in the immunoprecipitated protein intensities between P and E1 and E5 (**a**–**d**) and between P and I1 and I5 (**e**–**g**). Number of asterisks indicates level of statistical significance (*p < 0.05, **p < 0.01 and ***p < 0.001).

Some of the significant differences between the placebo and treatment groups were found on both treatment days (Fig. 5). The levels of relative autoantibody reactivity’s for apolipoprotein E (ApoE), clusterin, plasma protease C1 inhibitor, and serum amyloid P component (SAP) were all found to be decreased in etomoxir-treated animals, whereas antibody reactivity for serum albumin was found to be increased. The same changes were found in the control group for plasma protease C1 inhibitor and serum albumin levels. In IFN-β-treated animals, the autoantibody reactivity for complement component C9 and gelsolin were decreased, whereas the relative autoantibody reactivity for PLP was upregulated, and only antibody reactivity for complement component C9 was the same as that in control animals.

## Discussion

The clinical efficacy of the CPT1 inhibitor etomoxir was compared to that of the current first-line treatment IFN-β in an EAE model of MS. Animals treated with etomoxir on day 1 had a statistically significantly lower disease score at day 11 than animals receiving placebo, IFN-β on day 1 and IFN-β on day 5. Moreover, the group treated with etomoxir on day 5 obtained a significantly lower disease score than the IFN-β on day 1 treatment group. These lower disease scores obtained after etomoxir treatment indicate that blocking beta- oxidation in the mitochondria is a target for treating EAE and potentially MS. This result is consistent with the finding of Shriver and colleagues, who showed a beneficial effect of etomoxir treatment in an EAE model^29^. Furthermore, Bakshi *et al*. found that anti-lipid IgG antibodies were associated with the worsening of brain MRI measurements in MS patients^45^. Gonzalo *et al*. found significantly increased amounts of autoantibodies to lipoxidized proteins, indicating that lipid peroxidation is a pathogenic pathway in MS^46^. Regarding IFN-β-treated groups, Shirani *et al*. found that IFN-β treatment did not slow the progression of disability in MS patients, which is consistent with the findings of the present study^5^.

Western blot analysis showed weaker antibody signals following treatment with etomoxir on day 1 compared to the other treatments. This result could indicate a lower abundance of antibodies in the serum, which could be an effect of etomoxir treatment. As the blotting was directed against IgG antibodies, it is important to consider that the half-life of these antibodies is four to eight days in mice and could thus be similar in rats^47^. However, it is unlikely that such a decrease in antibody concentration would be substantial enough to have an obvious effect in late-treated animals. The high similarity between these potential autoantigens indicated that autoantibodies were present even in healthy animals, raising the question of why the autoimmune response was not active in control animals. This result could be due to low concentrations of antibodies, low affinity towards the antigen or an intact blood-brain barrier (BBB).

In the current study, the placebo group had significantly higher serum concentrations of antibodies against positive acute phase reactants (α1-acid glycoprotein, complement component C9, haptoglobin, ceruloplasmin, and plasma protease C1 inhibitor) and significantly lower serum concentrations of antibodies against negative acute phase reactants (transthyretin and α1-inhibitor-3) than the control group. These findings are consistent with the literature^48–54^. Furthermore, complement component C9 has been found to be directly correlated with demyelination of the spinal cord^55^. A previous study showed elevated levels of hemopexin during the progression of disease in the EAE model, which is consistent with our findings^56^. In this study, EAE was induced by MBP suspended in complete Freund’s adjuvant; therefore, an antibody response to MBP was expected and was used as a positive control in this model^57,58^. The autoantibody response to MBP was validated by ELISA. The intensities of serum antibodies in the placebo group were consistent with the fact that the placebo group obtained a high disease score, which implies that high levels of positive acute phase reactants and antigens and low levels of negative acute phase reactants are associated with EAE and possibly MS.

Furthermore, the two etomoxir-treated groups had significantly lower serum concentrations of antibodies against ApoE, clusterin, SAP, and plasma protease C1 inhibitor and a significantly higher serum concentration of anti-albumin antibody than the placebo group. Studies have shown an association between ApoE and MS^59,60^. Of note, Zhao *et al*. examined the effect of ApoE mimetic peptides in a mouse model of diffuse brain injury and found that ApoE mimetic peptides improved memory function and protected against neuronal apoptosis by inhibiting the ERK1/2 pathway and Bax expression. Moreover, malondialdehyde content was significantly decreased, and superoxide dismutase content was significantly increased^61^. Pang *et al*. showed that treatment with ApoE mimetic peptides in an experimental model of subarachnoid hemorrhage resulted in reduced degradation of the BBB, which further resulted in less severe brain edema and neuron apoptosis, increased cerebral glucose uptake, and improved neurological functions. Moreover, the peptides inhibited cyclophilin, nuclear factor-kappaB, interleukin-6, tumor necrosis factor-α, and interleukin-1 and thus inflammation and BBB disruption^62^. This finding could indicate that the significant reduction in serum autoantibodies reactivity for ApoE resulted in decreased inactivation of ApoE and thereby a lower degree of inflammation, apoptosis and breakdown of the BBB, and this effect could explain the significantly lower disease score in etomoxir-treated animals than in placebo-treated animals. Furthermore, based on observations by Pang and colleagues, blocking lipid metabolism by etomoxir could also have an effect on the level of ApoE and result in normalization of glucose metabolism in the brain. Clusterin has been found to be upregulated in acute and chronic plaques in MS^52^. A previous study indicated that clusterin could be a marker of neurodegeneration in AD and PD and that its expression is increased during progression of AD^63^. Moreover, clusterin and complement factors leak into the brain during BBB breakdown^64^. These findings raise the question of whether the significant reduction in the relative autoantibody reactivity for clusterin in etomoxir-treated animals could be the result of reduced degradation of the BBB. Moreover, clusterin facilitates the removal of apoptotic cells and thus diminishes autoimmune responses following cell death^65^. The lower levels of serum anti-clusterin antibodies and the lesser degree of disease in etomoxir-treated animals at day 1 and day 5 could indicate that clusterin facilitates the clearance of apoptotic cells. Ji *et al*. found that SAP might have a protective role in the development of EAE^66^. In support of their findings, Grant and colleagues found that treatment with amyloid β−40 or amyloid β-42 peptides reduced motor paralysis and brain inflammation in four different EAE models^67^. Furthermore, Kurnellas *et al*. showed that amyloidogenic peptides such as tau, amyloid-β A4 and SAP had anti-inflammatory properties, reduced serum levels of interleukin-6 and reduced paralysis in EAE^68,69^. This finding could indicate that the significant decrease in serum anti-SAP antibodies resulted in decreased inactivation of SAP and thereby a lesser degree of inflammation. A review by Levine indicated that serum albumin has two side effects in the brain^70^: anti-pathogenic properties because it is a target for ROS and pro-pathogenic properties because it is pro-inflammatory, and high levels of serum albumin can lead to seizure. Furthermore, serum albumin leaks into the brain during BBB breakdown and is taken up by macrophages and cleaved during the acute phase of MS. In most healthy individuals anti-albumin antibodies have been found^71^, and it was therefore expected to detect these antibodies in control animals. Interestingly, in the EAE-diseased animals anti-albumin antibodies were decreased. These results were validated by ELISA.

IFN-β-treated animals at days 1 and 5 had significantly lower serum concentrations of antibodies against complement component C9 and gelsolin than the placebo group. An earlier study found a direct correlation between the level of complement component C9 and demyelination of the spinal cord in an EAE model^55^. The lower serum antibody reactivity for complement component C9 could indicate that it is more active, which would result in demyelination and thereby a high disease score in IFN-β-treated animals^72^. Gelsolin is involved in myelin wrapping and is decreased in cerebrospinal fluid and serum in MS but elevated in brain tissue^73,74^. The low serum concentration of anti-gelsolin antibody could indicate that myelin wrapping in IFN-β-treated animals was affected. Furthermore, IFN-β-treated animals at days 1 and 5 had significantly higher serum concentrations of antibodies against PLP than placebo-treated animals, which is consistent with the higher disease score in IFN-β-treated animals. However, etomoxir-treated animals at day 5 also showed higher serum levels of antibodies against PLP than placebo-treated animals, highlighting a potential topic of interest for further investigation.

In conclusion, this study indicated that etomoxir is a more effective treatment for MBP-induced EAE than IFN-β and placebo, as it resulted in a significantly lower disease scores. Additionally, the results indicate that etomoxir-dependent inhibition of mitochondrial beta-oxidation in the EAE rat model is effective because of the regulation of serum autoantibodies against proteins such as ApoE, clusterin, SAP, and serum albumin, suggesting a role of these antigens in the progression of EAE and their potential use as diagnostic biomarkers for MS.

## Materials and Methods

### Experimental autoimmune encephalomyelitis rat model

Animal experiments were conducted according to NIH guidelines and were approved by the Danish National Committee for Ethics in Animal Experimentation (2015-15-0201-00647). Two-month-old female Lewis rats were obtained from Charles River Laboratories, Inc., Germany. The rats were housed under standardized conditions with 12 h light/dark cycles and free access to food and water. The rats were anesthetized with isoflurane (Baxter Laboratories) and then intradermally injected in the base of the tail with an emulsion consisting of 100 µg of MBP (Sigma-Aldrich) suspended in saline and complete Freund’s adjuvant (Becton Dickinson) and supplemented with 0.2 µg of *Mycobacterium tuberculosis* (Becton Dickinson). EAE was induced in 45 rats, and five rats were used as controls and thus were not EAE-induced. Rats were treated daily with either etomoxir (Meta-IQ ApS, Denmark) or IFN-β (Extavia, Aalborg University Hospital, Denmark) on the first or fifth day of induction. Etomoxir was heated to 37 °C and dissolved in olive oil. Etomoxir was subcutaneously administered every day at a dosage of 1 mg/kg. Interferon-β was subcutaneously administered every other day at a dosage of 200,000 IU. The placebo group received daily injections of olive oil. All rats were weighed and clinically scored on a daily basis according to a scale from zero to five^75^: zero, no clinical signs of EAE; one, limp tail; two, paresis of one or two hind limbs; three, unilateral hind leg paralysis; four, bilateral hind leg paralysis; and five, bilateral hind leg paralysis and incontinence or moribund. Moreover, animals were not permitted to lose more than 20% body weight compared to their weight at the time of EAE induction. Rats were euthanized. Then, blood samples were obtained by cardiac puncture, and collected blood samples were labeled according to the groups. Blood samples were used for Western blot, ELISA and immunoprecipitation.

### Western blot

#### Preparation of rat brain sample

Cerebellum from three-week-old Sprague-Dawley rats was isolated, and protein homogenates were prepared by transferring the cerebellum to a dissection buffer containing 10% sucrose (VWR), imidazole (Merck Millipore), EDTA (Sigma-Aldrich), Pefabloc (Sigma-Aldrich) and leupeptin (VWR). The mixture was homogenized (T10 basic ULTRA-TURAX homogenizer IKA®) for 20 sec; then, samples were centrifuged at 1000 g for 15 min. Supernatants were stored at −20 °C.

#### Sodium dodecyl sulfate-polyacrylamide gel electrophoresis

Brain homogenate samples were diluted in dissection buffer. Then, sample buffer (Bio-Rad) and 5% beta- mercaptoethanol (Sigma-Aldrich) were added to the brain homogenate solution at a 1:1 ratio. Samples were incubated for 15 min at 65 °C. Precision Plus Protein Standard Ladder (Bio-Rad) was used as a reference. Western blots of total rat brain tissue were separated on 4–15% Mini-PROTEAN^®^ TGX™ Precast Protein Gels (Bio-Rad) using a Mini-PROTEAN Tetra Cell system (Bio-Rad).

#### Blotting

Proteins were transferred to polyvinylidene fluoride membranes (Bio-Rad) by using a Trans-Blot Turbo Transfer System (Bio-Rad). Membranes were stained with 0.1% Ponceau S (MP Biomedicals; diluted in 1% acetic acid (VWR) and Milli-Q water) for 10 min, and gels were stained with Coomassie (Sigma-Aldrich) for 15 min. After washing three times in Milli-Q water, membranes and gels were respectively destained in 1% acetic acid in Milli-Q water for one hour and 10% ethanol, 7.5% acetic acid in Milli-Q water overnight, respectively. Membranes were cut vertically into lanes and blocked in 5% skimmed milk (VWR) diluted in 0.1% Tween- 20 (Sigma-Aldrich) in PBS (PBS-T) for one hour on a table shaker at room temperature. Then, membranes were washed three times in PBS-T and incubated overnight on a table shaker at 4 °C with sera (1:100; diluted in 1% BSA, 0.1% NaN_3_ in PBS-T) from healthy rats or EAE-induced rats that received placebo, etomoxir or IFN-β. After washing three times in PBS-T, membranes were incubated with rabbit anti-rat secondary antibody conjugated with HRP (cat#61-9520, Thermo Scientific) diluted in 5% skimmed milk in PBS-T (1:1000) for one hour on a table shaker at room temperature. Membranes were washed three times in PBS-T and subsequently incubated in Amersham ECL Prime Western blot Detection Reagent (GE Healthcare) for 1 min. Membranes were then visualized using an Odyssey Fc Imaging System (Li-Cor Biosciences) with LI-COR Image Studio™ software.

### Indirect Enzyme-Linked ImmunoSorbent Assay

Wells were coated with a protein concentration of 10 µg/ml of rat MBP (cat#2295, Sigma-Aldrich) or rat albumin (cat#A6414, Sigma-Aldrich) overnight at 4 °C. The coated wells were blocked by adding blocking buffer containing 0.1% Tween-20 (Sigma-Aldrich) and 5% skimmed milk (VWR) in PBS, and incubated for one hour at room temperature. The wells were washed three times in PBS. Afterwards, serum samples diluted in blocking buffer (1:100) were added and incubated for one hour at room temperature. At the same time, standards were prepared in a range of 1:10 to 1:100000 using rat anti-MBP (cat#ab7349, Abcam) and rabbit anti-albumin (cat#ab207327, Abcam) antibodies diluted in blocking buffer and incubated for one hour at room temperature. All samples and standards for MBP were run in triplicates and in duplicates for albumin. After incubation, all wells were washed three times in PBS. Secondary antibodies, rabbit anti-rat conjugated with HRP (cat#61-9520, Thermo Scientific) and goat anti-rabbit conjugated with HRP (cat#P0448, Dako) diluted in blocking buffer (1:2000), were added and incubated for one hour at room temperature. All wells were washed four times in PBS and then 3,3′5,5′-tetramethylbenzidine (Sigma-Aldrich) were added and incubated for 15 min at room temperature. Following incubation, stop solution containing MilliQ-water and 2 M H_2_SO_4_ (1:1) were added. Subsequently, the optical density was read at 450 nm using EnSpire Multimode Plate Reader. The concentration of autoantibodies was calculated using the standard curves in GraphPad Prism 7.

### Immunoprecipitation

#### Preclearing

A mixture of antibody buffer containing 1% BSA, 0.1% 2 M NaN_3_ in PBS-T and A/G magnetic beads (Pierce Protein Biology, Thermo Scientific) was prepared and transferred to a tube in a magnetic stand. Then, the supernatant was discarded, and the magnetic beads were collected. Antibody buffer was added to the magnetic beads mixture and vortexed. The mixture was then added to brain homogenate obtained from cerebellum of a three-week-old Sprague-Dawley rat and incubated on ice for one hour. Next, this solution was transferred to the magnetic stand, and the beads were collected, washed three times in antibody buffer and subsequently stored at 4 °C. The supernatant was collected, and additional magnetic beads were added and incubated on ice for 30 min. This step was repeated three times, and antibody buffer was added to the supernatant.

#### Immunoprecipitation

A total of 1 µl of serum from healthy rats and EAE-induced rats receiving placebo, etomoxir or IFN-β, was used for immunoprecipitation experiments. Precleared brain homogenate, which was the same for all groups, was mixed with serum from EAE animals and incubated on ice for one hour. Magnetic beads were added to each sample and incubated on ice for one hour. After incubation, samples were placed in a magnet stand to isolate the beads, which were washed three times in antibody buffer and then eluted by incubating in 120 mM sodium deoxycholate (SDC) in 50 mM triethylammonium bicarbonate (TEAB) (pH 8.5) for 10 min at 95 °C.

### Proteomics sample preparation

Proteomic sample preparation was performed according to a FASP digestion protocol by Bennike *et al*., in which samples are prepared with ethyl acetate phase inversion to facilitate surfactant removal^76^. The eluate was transferred to individual YM-10 kDa spin filters for digestion (Millipore, Billerica, MA, USA) and centrifuged at 14,000 g for 15 min at room temperature, which were the settings for all future performed centrifugation steps. Protein disulfide bonds were reduced with 12 mM tris(2-carboxyethyl)phosphine (Thermo Scientific) and alkylated with 50 mM chloroacetamide (Sigma-Aldrich) for 30 min at 37 °C, followed by centrifugation. Reducing and alkylating agents were dissolved in 120 mM SDC/50 mM TEAB (pH 8.5). In preparation for digestion, 10 mM CaCl and 50 mM TEAB buffer was added to the spin filter and centrifuged. A 1:25 (w/w) chymotrypsin:protein ratio dissolved in digestion buffer was added to the spin filter, and the samples were digested overnight at 37 °C. Flow-through, containing the peptides, was retrieved by addition of 50 mM TEAB buffer and centrifugation. To facilitate SDC removal, a phase separation was performed with 3:1 (v/v) ethyl acetate:sample and acidified by addition of trifluoroacetic acid (TFA) to a final concentration of 0.5%. Total phase separation was achieved by 2 min agitation followed by centrifugation. The aqueous phase was collected and vacuum centrifuged overnight, and the dry peptide product was stored at −80 °C until analysis.

### Mass spectrometry analysis

Peptides were resuspended in a solution containing 2% acetonitrile (ACN), 0.1% formic acid (FA) and 0.1% TFA, and then, peptides were briefly sonicated. Five micrograms of total peptide material were analyzed per liquid chromatography–mass spectrometry analysis. Samples were analyzed using a UPLC-nanoESI MS/MS setup with a NanoRSLC system (Dionex). The system was coupled online with an emitter for nanospray ionization (New Objective PicoTip 360-20-10) to a Q Exactive HF mass spectrometer (Thermo Scientific). The peptide material was loaded onto a 2-cm trapping reversed-phase Acclaim PepMap RSLC C18 column (Dionex) and separated using an analytical 75-cm reversed-phase Acclaim PepMap RSLC C18 column (Dionex). Both columns were kept at 60 °C. The sample was eluted with a gradient of 90% solvent A (0.1% FA, 0.1% TFA) and 10% solvent B (0.1% FA, 0.1% TFA in ACN), which was increased to 7% solvent B on a 1-min ramp gradient at a constant flow rate of 300 nL/min. Subsequently, the gradient was raised to 30% solvent B on a 45-min ramp gradient. The mass spectrometer was operated in positive mode, selecting up to 20 precursor ions with a mass window of m/z 1.6 based on highest intensity for higher-energy collisional dissociation (HCD) fragmenting at a normalized collision energy of 27. Selected precursors were dynamically excluded for fragmentation for 30 sec.

### Protein identification and quantitation analysis

A label-free relative quantitation analysis was performed using MaxQuant 1.5.7.4 software. Raw files were searched against the *Rattus norvegicus* UniProt database (proteome ID UP000002494)^77,78^. All standard settings were employed with carbamidomethylation (C) as a static peptide modification and deamidation (NQ), oxidation (M), formylation (N-terminal and K), and protein acetylation (N-terminal) as variable modifications. The output contained a list of proteins identified below 1% false discovery rate, and their abundances were further filtered and processed using Perseus v1.5.6.0 platform. All reverse hits that identified proteins were removed from further analysis, and the data were log2-transformed in order to approximate normal distribution. Two or more unique peptides were required for protein quantitation. Additionally, a non-zero quantitation value in at least 70% of the samples in one of the groups was required for the quantifiable proteins. A Venn diagram was generated using FunRich software to compare the number of proteins that were present in 70% of the samples in each group^79^. The mass spectrometry proteomics data have been deposited to the ProteomeXchange Consortium via the PRIDE partner repository with the dataset identifier PXD008835^80,81^.

### Applied statistics

Disease score data were analyzed by unpaired t-tests. ELISA data were quantified by interpolating samples according to a standard curve and groups were analyzed by one-way ANOVA. Quantifiable proteins identified in the different groups were compared in a Venn diagram, and to investigate differences in the expression of proteins between the groups, unpaired two-tailed t-tests were performed. All data are presented as the mean ± SEM. P values of 0.05 were used as a cut-off.

## Electronic supplementary material

Supplementary Figure S1
