# Supplementary material for: Identification of brain antigens recognized by autoantibodies in experimental autoimmune encephalomyelitis-induced animals treated with etomoxir or interferon-β
Source: Sci Rep. 2018 May 4;8:7092. doi: 10.1038/s41598-018-25391-y (PMC5935685; doi:10.1038/s41598-018-25391-y)
Supplement: Supplementary file 1 — Supplementary Figure S1 [file 41598_2018_25391_MOESM1_ESM.pdf]

Supplementary Figure S1

Identification of brain antigens recognized by autoantibodies in experimental autoimmune encephalomyelitis-induced animals treated with etomoxir or interferon-β

Anne Skøttrup Mørkholt[1,¶], Kenneth Kastaniegaard[1,¶], Michael Trabjerg[1], Gopana Gopalasingam[1], Wanda Niganze[1], Agnete Larsen[2], Allan Stensballe[1], Søren Nielsen[1], and John Dirk Nieland[1,*]

	Compared to placebo										Compared to control									
	C		E1		E5		I1		I5		P		E1		E5		I1		I5	
**Protein names:**	Ratio	P value	Ratio	P value	Ratio	P value	Ratio	P value	Ratio	P value	Ratio	P value	Ratio	P value	Ratio	P value	Ratio	P value	Ratio	P value
T-kininogen 1	▼ -1,87	0,00	▼ -0,04	0,92	▼ -0,52	0,37	▲ 0,33	0,19	▼ 0,00	0,99	▲ 1,87	0,00	▲ 1,83	0,01	▲ 1,35	0,12	▲ 2,20	0,00	▲ 1,87	0,00
Myelin basic protein	▼ -1,89	0,03	▼ -0,85	0,19	▼ -0,77	0,26	▼ -0,74	0,24	▼ -0,17	0,81	▲ 1,89	0,03	▲ 1,04	0,06	▲ 1,13	0,10	▲ 1,15	0,07	▲ 1,72	0,04
Alpha-1-acid glycoprotein	▼ -3,65	0,00	▼ -0,40	0,43	▼ -0,43	0,36	▲ 0,36	0,37	▼ -0,22	0,63	▲ 3,65	0,00	▲ 3,25	0,00	▲ 3,22	0,00	▲ 4,00	0,00	▲ 3,42	0,00
Transthyretin	▲ 0,96	0,05	▼ -0,26	0,47	▼ -0,07	0,86	▼ -0,41	0,18	▼ -0,35	0,45	▼ -0,96	0,05	▼ -1,22	0,01	▼ -1,03	0,05	▼ -1,38	0,00	▼ -1,31	0,04
T-kininogen 2	▼ -2,54	0,00	▼ -0,17	0,74	▼ -0,59	0,30	▲ 0,23	0,35	▼ -0,09	0,76	▲ 2,54	0,00	▲ 2,36	0,01	▲ 1,94	0,03	▲ 2,77	0,00	▲ 2,44	0,00
Serine protease inhibitor A3N	▼ -1,87	0,02	▼ -0,81	0,22	▼ -0,76	0,16	▼ -0,22	0,63	▼ -0,58	0,26	▲ 1,87	0,02	▲ 1,06	0,21	▲ 1,12	0,08	▲ 1,65	0,00	▲ 1,29	0,04
Ceruloplasmin	▼ -1,35	0,01	▼ -0,18	0,63	▼ -0,31	0,39	▼ -0,34	0,30	▼ -0,28	0,38	▲ 1,35	0,01	▲ 1,17	0,03	▲ 1,04	0,04	▲ 1,01	0,02	▲ 1,07	0,02
Alpha-1-inhibitor 3	▲ 1,09	0,01	▼ -0,24	0,59	▼ -0,39	0,25	▼ -0,41	0,16	▼ -0,30	0,39	▼ -1,09	0,01	▼ -1,33	0,05	▼ -1,48	0,01	▼ -1,50	0,00	▼ -1,38	0,01
Hemopexin	▼ -1,05	0,01	▼ -0,48	0,44	▼ -0,62	0,31	▼ -0,05	0,88	▼ -0,01	0,98	▲ 1,05	0,01	▲ 0,57	0,51	▲ 0,44	0,61	▲ 1,01	0,02	▲ 1,05	0,01
Neurocan core protein	▼ -0,66	0,02	▼ -0,28	0,49	▼ -0,06	0,88	▼ -0,06	0,87	▲ 0,17	0,72										
Serum albumin	▲ 1,07	0,00	▲ 1,04	0,00	▲ 0,60	0,05	▲ 0,21	0,41	▲ 0,31	0,28	▼ -1,07	0,00	▼ -0,03	0,92	▼ -0,47	0,19	▼ -0,87	0,01	▼ -0,76	0,04
Plasma protease C1 inhibitor	▼ -1,78	0,04	▼ -1,54	0,01	▼ -1,47	0,02	▼ -1,38	0,02	▼ -0,90	0,08	▲ 1,78	0,04	▲ 0,24	0,72	▲ 0,31	0,65	▲ 0,40	0,54	▲ 0,88	0,13
Keratin, type II cytoskeletal 1	▲ 2,18	0,00	▲ 1,19	0,20	▲ 1,41	0,02	▲ 2,57	0,01	▲ 0,62	0,53	▼ -2,18	0,00	▼ -0,99	0,38	▼ -0,77	0,21	▲ 0,39	0,71	▼ -1,57	0,23
Complement component C9	▼ -2,19	0,04	▼ -0,96	0,20	▼ -1,05	0,10	▼ -1,48	0,05	▼ -1,40	0,04	▲ 2,19	0,04	▲ 1,22	0,21	▲ 1,13	0,11	▲ 0,71	0,33	▲ 0,79	0,16
Haptoglobin	▼ -2,64	0,00	▼ -0,77	0,14	▼ -0,47	0,18	▼ -0,54	0,11	▼ -0,70	0,05	▲ 2,64	0,00	▲ 1,87	0,01	▲ 2,18	0,00	▲ 2,10	0,00	▲ 1,95	0,00
Apolipoprotein C-III	▼ -0,39	0,38	▼ -1,96	0,00	▼ -0,84	0,09	▲ 0,25	0,48	▼ -0,34	0,38	▲ 0,39	0,38	▼ -1,57	0,01	▼ -0,45	0,35	▲ 0,64	0,08	▲ 0,05	0,90
Beta-2-microglobulin	▼ -1,18	0,10	▼ -1,13	0,04	▼ -0,09	0,89	▲ 0,04	0,94	▼ -1,07	0,09	▲ 1,18	0,10	▲ 0,05	0,91	▲ 1,09	0,05	▲ 1,22	0,07	▲ 0,10	0,87
Protein AMBP	▲ 1,02	0,32	▲ 1,87	0,01	▲ 1,40	0,07	▲ 0,93	0,17	▲ 1,05	0,11										
Apolipoprotein E	▼ -0,90	0,11	▼ -1,21	0,02	▼ -1,18	0,02	▼ -0,44	0,26	▼ -0,66	0,12										
Clusterin	▲ 0,00	1,00	▼ -1,37	0,05	▼ -1,99	0,01	▼ -0,20	1,00	▼ -0,67	0,31										
Serum amyloid P-component	▼ -0,08	0,90	▼ -1,41	0,03	▼ -1,56	0,01	▼ -0,85	0,13	▼ -0,52	0,30										
Hemoglobin subunit alpha-1/2	▲ 0,23	0,59	▲ 0,44	0,28	▼ -0,82	0,04	▼ -0,20	0,68	▼ -0,27	0,49	▼ -0,23	0,59	▲ 0,21	0,67	▼ -1,05	0,04	▼ -0,43	0,53	▼ -0,50	0,32
Hemoglobin subunit beta-1	▼ -0,42	0,36	▼ -0,26	0,47	▼ -1,31	0,01	▼ -0,14	0,74	▼ -0,70	0,09										
Hemoglobin subunit beta-2	▼ -0,61	0,32	▼ -0,56	0,23	▼ -1,28	0,04	▼ -0,46	0,33	▼ -0,58	0,19										
Apolipoprotein C-I	▼ -0,65	0,47	▼ -1,47	0,09	▼ -1,54	0,03	▼ -1,17	0,12	▼ -1,14	0,17	▲ 0,65	0,47	▼ -0,81	0,10	▼ -0,88	0,00	▼ -0,52	0,23	▼ -0,48	0,31
Myelin proteolipid protein	▲ 0,22	1,00	▲ 2,72	0,06	▲ 3,24	0,03	▲ 3,68	0,00	▲ 3,05	0,02										
Complement C3	▼ -0,72	0,15	▼ -0,42	0,29	▼ -0,43	0,21	▼ -0,77	0,04	▼ -0,69	0,08										
Fibronectin	▼ -0,85	0,50	▼ -1,57	0,11	▼ -1,07	0,26	▼ -2,30	0,02	▼ -1,07	0,38										
Carboxylesterase 1C	▲ 1,03	0,20	▼ -0,24	0,67	▼ -0,48	0,44	▼ -1,70	0,02	▼ -0,61	0,30	▼ -1,03	0,20	▼ -1,27	0,02	▼ -1,51	0,02	▼ -2,73	0,00	▼ -1,64	0,01
Neural cell adhesion molecule 1	▼ -0,10	0,82	▲ 0,01	0,98	▲ 0,52	0,25	▼ -0,82	0,05	▲ 0,37	0,21										
Murinoglobulin-1	▲ 0,82	0,11	▼ -0,43	0,27	▼ -1,08	0,07	▼ -0,89	0,04	▼ -0,69	0,11	▼ -0,82	0,11	▼ -1,25	0,01	▼ -1,90	0,02	▼ -1,71	0,00	▼ -1,52	0,01
Keratin, type I cytoskeletal 10	▲ 2,17	0,21	▲ 0,83	0,28	▲ 2,41	0,12	▲ 3,43	0,01	▲ 1,98	0,10										
Amphoterin-induced protein 1	▲ 0,64	0,52	▲ 1,10	0,16	▲ 1,47	0,07	▲ 1,72	0,03	▲ 1,10	0,15										
Gelsolin	▲ 0,31	0,66	▼ -0,15	0,75	▼ -0,80	0,14	▼ -1,58	0,02	▼ -1,24	0,03	▼ -0,31	0,66	▼ -0,45	0,38	▼ -1,11	0,11	▼ -1,89	0,03	▼ -1,55	0,03
Apolipoprotein A-IV	▲ 0,25	0,76	▼ -0,98	0,12	▼ -0,60	0,35	▼ -0,95	0,15	▼ -1,63	0,03	▼ -0,25	0,76	▼ -1,23	0,02	▼ -0,84	0,06	▼ -1,20	0,06	▼ -1,87	0,02
Heparin cofactor 2	▼ -0,97	0,16	▼ -0,97	0,28	▼ -2,17	0,06	▼ -1,13	0,07	▼ -1,66	0,02										
Apolipoprotein A-II											▼ -0,94	0,35	▼ -1,53	0,02	▼ -1,03	0,02	▼ -0,75	0,01	▼ -1,19	0,01
Serine protease inhibitor A3L											▼ -0,78	0,18	▼ -1,44	0,02	▼ -1,51	0,01	▼ -1,42	0,00	▼ -1,57	0,04
Rab3 GTPase-activating protein non-catalytic subunit											▼ -1,25	0,10	▼ -1,41	0,03	▼ -2,01	0,04	▼ -0,81	0,13	▼ -1,54	0,03
Voltage-dependent anion-selective channel protein 1											▲ 0,97	1,00	▲ 0,97	0,00	▲ 0,65	0,10	▲ 0,66	0,02	▲ 0,77	0,04
V-type proton ATPase 16 kDa proteolipid subunit											▼ -0,05	0,94	▲ 0,49	0,20	▲ 1,33	0,05	▼ -0,61	0,40	▲ 0,07	0,92
Alpha-2-HS-glycoprotein											▼ -1,29	0,11	▼ -1,54	0,07	▼ -1,65	0,02	▼ -1,60	0,01	▼ -1,42	0,02
Ral GTPase-activating protein subunit alpha-1											▲ 0,63	0,54	▲ 1,24	0,12	▲ 0,89	0,20	▲ 2,14	0,00	▲ 0,67	0,45
Neurofilament light polypeptide											▲ 1,28	0,07	▲ 1,02	0,11	▲ 1,01	0,24	▲ 1,21	0,01	▲ 0,98	0,33
Beta-2-glycoprotein 1											▼ -0,42	0,42	▼ -0,71	0,26	▼ -0,66	0,29	▼ -1,34	0,04	▼ -0,97	0,14
Guanine nucleotide-binding protein subunit beta-2											▼ -0,27	0,67	▼ -0,53	0,29	▼ -0,08	0,86	▼ -0,97	0,02	▼ -0,27	0,54
60S ribosomal protein L23a											▼ -0,83	0,32	▼ -1,12	0,09	▼ -0,13	0,86	▼ -1,12	0,04	▼ -0,11	0,87
Leucine-rich repeat protein SHOC-2											▼ -1,11	0,05	▼ -1,08	0,15	▼ -1,49	0,13	▼ -1,58	0,01	▼ -1,12	0,08
Serine protease inhibitor A3K											▼ -0,86	0,18	▼ -1,17	0,07	▼ -0,92	0,18	▼ -1,80	0,03	▼ -1,22	0,03
Corticosteroid-binding globulin											▼ -0,88	0,28	▼ -0,74	0,19	▼ -1,05	0,08	▼ -1,76	0,02	▼ -1,32	0,02
Ig kappa chain V region S211											▲ 1,50	0,14	▲ 1,06	0,16	▲ 0,86	0,19	▲ 1,15	0,14	▲ 2,00	0,05
Retinol-binding protein 4											▼ -0,13	0,85	▼ -0,55	0,29	▼ -0,92	0,12	▼ -0,72	0,15	▼ -1,20	0,02
Peptidyl-prolyl cis-trans isomerase A											▼ -1,18	0,19	▼ -0,36	0,58	▼ -0,70	0,37	▼ -0,39	0,44	▼ -1,37	0,05
